# Supplementary figures and images for: Distribution, organization and expression of genes concerned with anaerobic lactate utilization in human intestinal bacteria
Source: Microb Genom. 2022 Jan 25;8(1):000739. doi: 10.1099/mgen.0.000739 (PMC8914356; doi:10.1099/mgen.0.000739)

**Figure S1.** Illustrative schematic figure showing the transcriptomic experimental workflow

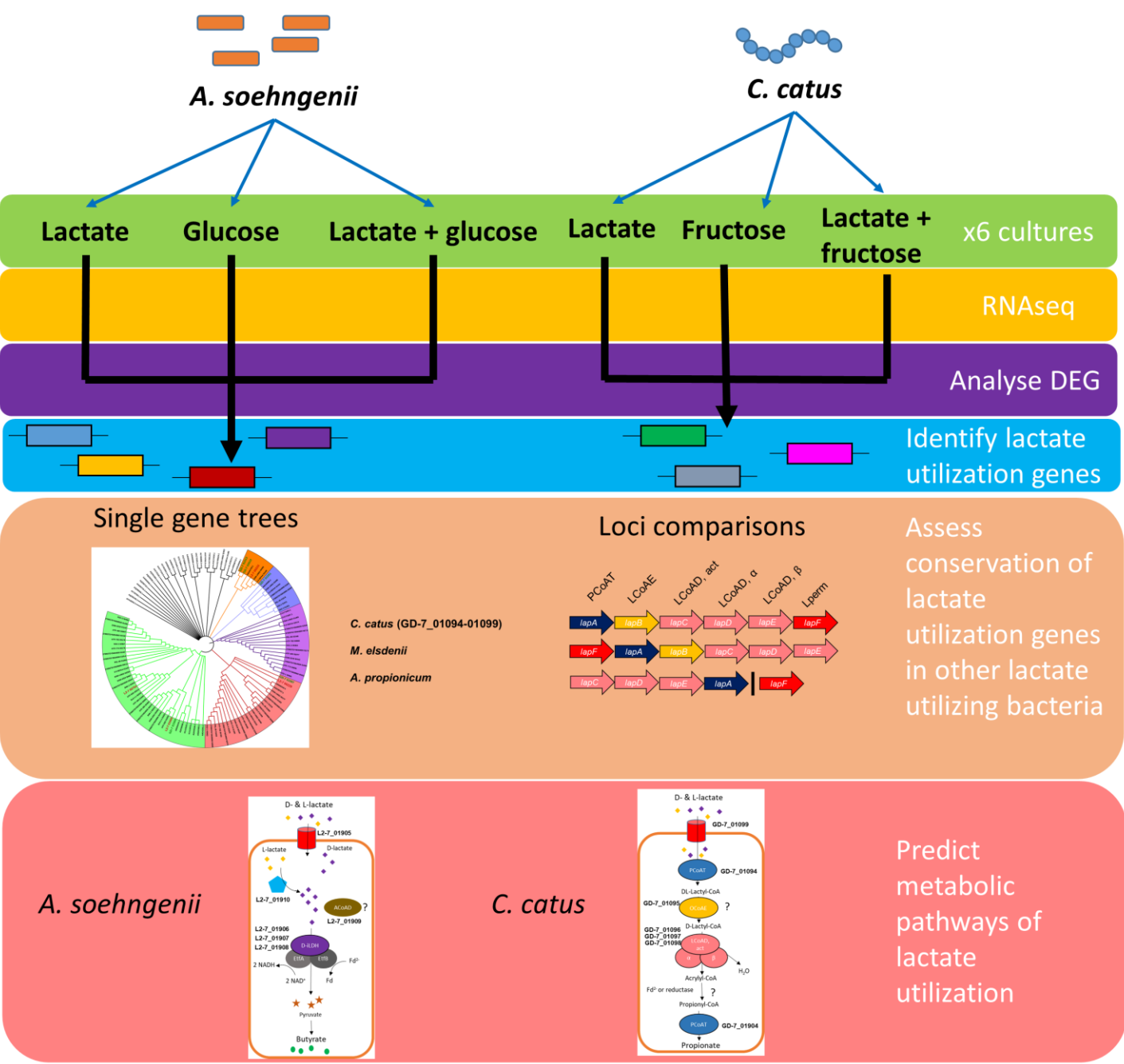

Supplement: Supplementary material 1 [file mgen-8-0739-s001.pdf]
